# An Intelligent Clinical Decision Support System for Patient-Specific Predictions to Improve Cervical Intraepithelial Neoplasia Detection

**DOI:** 10.1155/2014/341483

**Published:** 2014-04-09

**Authors:** Panagiotis Bountris, Maria Haritou, Abraham Pouliakis, Niki Margari, Maria Kyrgiou, Aris Spathis, Asimakis Pappas, Ioannis Panayiotides, Evangelos A. Paraskevaidis, Petros Karakitsos, Dimitrios-Dionyssios Koutsouris

**Affiliations:** ^1^Biomedical Engineering Laboratory, School of Electrical and Computer Engineering, National Technical University of Athens, Iroon Politechniou 9, 15773 Zografou Campus, Athens, Greece; ^2^Institute of Communication and Computer Systems, National Technical University of Athens, Iroon Politechniou 9, 15773 Zografou Campus, Athens, Greece; ^3^Department of Cytopathology, School of Medicine, University General Hospital “ATTIKON”, University of Athens, Rimini 1, 12462 Athens, Greece; ^4^West London Gynaecological Cancer Center, Queen Charlotte's and Chelsea, Hammersmith Hospital, Imperial Healthcare NHS Trust, London W12 0HS, UK; ^5^Division of Surgery and Cancer, Faculty of Medicine, Imperial College, London W12 0NN, UK; ^6^3rd Department of Obstetrics and Gynecology, University General Hospital “ATTIKON”, School of Medicine, University of Athens, Rimini 1, 12462 Athens, Greece; ^7^2nd Department of Pathology, University General Hospital “ATTIKON”, School of Medicine, University of Athens, Rimini 1, 12462 Athens, Greece; ^8^Department of Obstetrics and Gynecology, University Hospital of Ioannina, St. Niarchou Str, 45500 Ioannina, Greece

## Abstract

Nowadays, there are molecular biology techniques providing information related to cervical cancer and its cause: the human Papillomavirus (HPV), including DNA microarrays identifying HPV subtypes, mRNA techniques such as nucleic acid based amplification or flow cytometry identifying E6/E7 oncogenes, and immunocytochemistry techniques such as overexpression of p16. Each one of these techniques has its own performance, limitations and advantages, thus a combinatorial approach via computational intelligence methods could exploit the benefits of each method and produce more accurate results. In this article we propose a clinical decision support system (CDSS), composed by artificial neural networks, intelligently combining the results of classic and ancillary techniques for diagnostic accuracy improvement. We evaluated this method on 740 cases with complete series of cytological assessment, molecular tests, and colposcopy examination. The CDSS demonstrated high sensitivity (89.4%), high specificity (97.1%), high positive predictive value (89.4%), and high negative predictive value (97.1%), for detecting cervical intraepithelial neoplasia grade 2 or worse (CIN2+). In comparison to the tests involved in this study and their combinations, the CDSS produced the most balanced results in terms of sensitivity, specificity, PPV, and NPV. The proposed system may reduce the referral rate for colposcopy and guide personalised management and therapeutic interventions.

## 1. Introduction

Cervical cancer is the third most common cancer and the fourth leading cause of cancer death in females worldwide [1]. Cervical cancer is known to be caused almost always by human papillomavirus (HPV) infection which is the commonest sexually transmitted infection worldwide. However, the presence of HPV does not always lead to disease [2]. About 100 types of HPV virus have been identified that can infect humans. Among them, at least 15 are oncogenic and thus can cause cancer of the cervix [3, 4]. Improved understanding of HPV infection and the natural history of cervical neoplasia have resulted in the addition of the HPV DNA test along with the Pap test.

From the meta-analysis of the most authoritative published studies [5–8] it can be concluded that the sensitivity of Pap test combined with the HPV DNA test is higher than the sensitivity of each individual method. This observation suggests that the two methods complement each other effectively. In contrast, the specificity of the Pap test combined with the HPV DNA test was lower than the ratings of the two methods separately as they differ in sensitivity and specificity [9, 10]. Regarding the positive predictive value (PPV) the findings are equivocal: some studies report that the values of PPV were similar for each method separately and for their combination, while others report smaller values of PPV for their combination. As expected, the negative predictive value (NPV) of HPV DNA test in conjunction with the Pap test was high and some studies report values of almost 100%.

In the recent years, new technologies for cervical cancer detection have been promoted to physicians and the public. Some studies proposed the shift from DNA detection to mRNA identification of the viral E6/E7 oncogenes that are linked to oncogenic activation. Among them, mRNA typing with nucleic acid sequence based amplification (NASBA) [11–13] and flow cytometry (mRNA-Flow-FISH) techniques for E6/E7 HPV mRNA detection have been enrolled in cancer and precancerous lesions' detection with promising results in increasing PPV and reducing unnecessary recalls and referrals to colposcopy [14–18]. At the same time, it seems that the immunocytochemical detection of genetic effects such as overexpression of p16 is a methodology which can increase the diagnostic accuracy of the Pap test [19, 20].

Several published studies in the literature are attempting to clarify the role of each technique as a unique test to substitute or replace the Pap test [5–8, 11, 14–24]. By the detailed analysis of the published studies it can be concluded that the performance of the methods under control differ significantly, affected by the disease incidence and the prevalence of HPV infection in the population study group, resulting in that the individual application of one method, even if it offers a level of protection, does not reliably determine the risk of each individual woman.

Advances in the areas of computer science and artificial intelligence allow the development of computer systems that support clinical diagnosis or therapeutic and treatment decisions based on individualised patient data [25, 26]. Clinical decision support systems (CDSSs) aim to codify and strategically manage biomedical knowledge to handle challenges in clinical practice using mathematical modelling tools, medical data processing techniques, and artificial intelligence methods. CDSSs cover a wide range of applications, from diagnosis' support to modelling the probability of occurrence of various diseases or the efficiency of alternative therapeutic schemes. To do so, they are using not only individual patient data but also data on risk factors and efficiency of available therapeutic schemes stored in databases. CDSSs are based on statistical analysis methods, such as regression analysis, or artificial intelligence techniques, such as artificial neural networks (ANNs) and pattern recognition techniques [27]. These can be used in order to extract hidden information with essential clinical value from large datasets. Based on complex algorithms, CDSSs may combine in a nonlinear complex way a number of characteristics, for example, data related to the patient (epidemiologic data, medical history, etc.), data related to the disease (examinations' results, biomarkers, course of the disease, etc.), or data related to the treatment (drug selection, drug doses, etc.). In this way, CDSSs provide clinicians with patient-specific assessments or recommendations to aid clinical decision making, or, even more, to provide predictions of diagnostic or prognostic outcomes.

Regarding cervical cancer, an intelligent decision making system may support physicians to improve the selection of protocols for monitoring, diagnosing, and treating women with intraepithelial lesions or cervical cancer or even support the rational selection and the patient-specific follow-up decision making for women who have been treated for high-grade lesions. The majority of published studies, regarding intelligent systems for cervical cancer support, are concerned about computer aided diagnosis systems based on either cytology or colposcopy image analysis [28–31]. On the other hand, various papers have been published in the past few years concerning bioinformatics' CDSSs based on ANNs for cancer improved detection, treatment, and follow-up support [32–37]. To the best of our knowledge, however, a similar bioinformatics intelligent CDSS for supporting and improving cervical cancer detection and triage, like the proposed system, has not been reported in the literature.

This study aims to investigate the potential role of a novel intelligent bioinformatics CDSS which intelligently combines the results of various diagnostic techniques used in the modern cytopathology laboratory in order to provide clinicians with patient-specific predictions of diagnostic or prognostic outcomes and thus to identify women at true risk of developing cervical cancer. The preliminary results suggest that the proposed system may improve the accuracy of diagnosis and in comparison to other combinatorial approaches produces the most balanced results in terms of specificity, sensitivity, PPV, and NPV.

## 2. Materials and Methods

### 2.1. Clinical Data

Data have been collected randomly from women enrolled in a research project conducted by the Department of Cytopathology of the Medical School of Athens University (“ATTIKON” University Hospital) and the Department of Obstetrics and Gynaecology of the University Hospital of Ioannina. Our study has been approved by the Bioethics Committee of the “ATTIKON” University Hospital and the Bioethics Committee of the University Hospital of Ioannina. Participating women had signed an informed patient consent (ICON) form allowing the use of their epidemiologic, diagnostic, and molecular data for the needs of the system's development and research. The clinical data (molecular examinations' results, cytological diagnoses, histological examination of biopsies, visit number and date, patient age, etc.) have been registered and stored into a database which has been developed for the research project purposes. For the processing of the data and algorithm training and testing, anonymised data were extracted from the database.

The study was carried out on ThinPrep LBC specimens obtained from women referred for colposcopy for two reasons: (1) either because they had a previous abnormal Pap test, or (2) they volunteered to participate in the study and accepted a colposcopical examination as well as the application of various biomarkers on their biological material even if they had a normal Pap test (e.g., women with HPV). In case that a negative Pap test was followed by negative colposcopy, no biopsy was taken and the case was considered as clinically negative. The smears were routinely prepared for cytological examination and the remaining material was used for evaluation of additional biomarkers (reflex tests). The cytology was assessed by experienced cytopathologists. Biopsies were obtained from samples during colposcopy and/or from surgical specimens through conization and were fixed and prepared according to standard histopathology protocols. All cases except the clinically negative ones were diagnosed by a single expert histopathologist as daily routine diagnosis and the evaluation of the biopsy was blinded from the results of cytology and other ancillary tests. The histopathologist uses as standard procedure p16 test (CINtec) in all histological material from the cervix.

The patients' database includes more than 5,000 patients with more than 10,000 tests' series due to follow-up cases. Each of these series includes the following tests: cytology according to the revised Bethesda classification (TBS2001 system) [38, 39]; HPV DNA typing using the CLART HUMAN PAPILLOMAVIRUS 2 (GENOMICA) that allows simultaneous detection of 35 different HPV genotypes (both high and low risk) by PCR amplification of a fragment within the highly conserved L1 region of the virus [40]; NASBA assays [41] (NucliSENS EasyQ HPV v1.0) that are used for the identification of E6/E7 mRNA of the HPV types 16, 18, 31, 33, and 45; the PermiFlow (Invirion Diagnostics, LLC, Oak Brook, IL) that allows the identification of E6/E7 mRNA expression of high-risk HPV using flow cytometry technique [17]; and finally the immunocytochemical expression of p16 using the CINtec Cytology Kit [42]. All these examinations produce results that can be used in a classification process and they provide assessments of the clinical cytological sample as a whole and not of individual cells.

Cytological findings of each patient were interpreted according to the Bethesda classification system and were classified as follows: (a) within normal limits (WNL); (b) atypical squamous cells of undetermined significance (ASCUS); (c) low-grade squamous intraepithelial lesion (LSIL); (d) high-grade squamous intraepithelial lesion (HSIL); (e) squamous cell carcinoma (SCC) or adenocarcinoma (Adeno-Ca). Regarding the HPV DNA test, for which the cytology laboratory is accredited by WHO and is proficient for the specific technique, we considered high-risk (HR) HPV types as HPV types 16, 18, 26, 31, 33, 35, 39, 45, 51, 52, 53, 56, 58, 59, 66, 68, 73, 82, and 85; and low-risk (LR) HPV types as HPV types 6, 11, 40, 42, 43, 44, 54, 61, 62, 70, 71, 72, 81, 83, 84, and 89 [3, 4]. It is well known that the probability of a low-risk subtype to cause cervical lesions is very small; however, the specific HPV DNA test is simultaneously identifying both high-risk and low-risk HPV subtypes and thus we used all available typing results during the development of the system, in order to evaluate its performance based on all available information.

For the cases that had histological outcome, the histological diagnosis was used as the golden standard. Random biopsies were not obtained in clinically negative cases, which are defined as cases that had negative cytology and additionally negative colposcopy; for these cases it is not allowed by the ethical committee to have a sample for histological examination. These cases may introduce a small bias in the interpretation of the cytology performance. A cervical biopsy was performed if Pap test revealed ASCUS and above cytological categories (ASCUS+) or there was a visible lesion upon colposcopy. Biopsy was performed by experienced colposcopists (in practice for more than 10 years) as part of the study protocol. The three-tiered cervical intraepithelial neoplasia (CIN) grading system was used for histological diagnosis; thus the cases with histology were classified as follows: (a) without evidence of malignancy (negative histology); (b) cervical intraepithelial neoplasia grade I (CIN1); (c) cervical intraepithelial neoplasia grade II or III (CIN2/3); (d) squamous cell carcinoma (SCC) or adenocarcinoma (Adeno-Ca).

From the more than 10,000 tests' series, 740 cases that were fully completed were selected for examination in this study and were further analysed (Table 1). Cases with one or more missing or invalid/inadequate tests' results were excluded from the study.

For each of the 740 cases, a feature set consisting of 46 variables derived by the applied tests was created. The result of the cytological examination has been used according to the Bethesda system. Results of the HPV DNA test examination were expressed as 35 individual variables (either positive or negative), one for each HPV DNA genotype. For the EasyQ test (NASBA) we used the result for each individual HPV type (16, 18, 31, 33, and 45). The result of the PermiFlow test (FLOW) was used as positive or negative, as was the result of the immunocytochemical expression of p16. Additionally, other variables expressing the HPV DNA test results were added; for instance, the existence of high-risk or low-risk types was expressed as either positive or negative.  Table 2 shows in detail the 46 independent variables/features that were collected for each case.

According to the final outcome (the histological examination or a clinically negative result), each of the 740 cases was classified into the following classes: class 1: negative or clinically negative, class 2: CIN1, class 3: CIN2 or CIN3 (CIN2/3), and class 4: cancer (SCC or Adeno-Ca).

### 2.2. Feature Selection

The first step of our study was to employ feature selection algorithms in order to identify which of the 46 aforementioned features contributes most to the prediction of the underlying condition of each woman, that is, the class of each case. The feature selection (FS) [43] problem in pattern recognition may be stated as follows: given a set of *N* features, find the best subset consisting of *l* features that contribute most to classification/prediction accuracy.

Feature subset selection algorithms can be classified into two main categories: the filter approach and the wrapper approach. In the filter approach the FS is done independently of the learning algorithm of a classifier. A class separability measuring criterion *C*(*k*) is adopted in order to rank all the features. The value of the criterion *C*(*k*) is computed for each of the features *k* = 1,2,…*m*. Features are then ranked in the order of descending values of *C*(*k*). The *l* features corresponding to the *l* best values of the specific criterion are selected to form the best subset. In wrapper type methods, the FS is “wrapped” around a learning method: the usefulness of a feature subset is directly judged by the estimated accuracy of a trained classifier. For high-dimensional datasets, wrapper methods are far too computationally expensive to be used because each feature subset considered must be evaluated with the trained classifiers. For this reason, wrapper methods will not be considered in this study.

In order to perform the FS task, we combined two different filter methods [44]. The area between the empirical receiver operating characteristic (ROC) curve and the random classifier slope has been proposed as a class separability measuring criterion [43]. As presented in [43], it measures the overlap between the probability density functions describing the data distribution of a feature in two classes. This criterion serves as a measure of the class discrimination capability of a specific feature. The second filter method we applied is the recently proposed minimum redundancy-maximum relevance (MRMR) feature selection framework, a mutual information based methodology, which has proved to be one of the best filter methods [45]. Both methods return the best feature subset for a selected value *l*.

During feature ranking using the ROC FS method, we applied an ad hoc cross correlation technique that incorporates correlation information into the ranking procedure so as to avoid the existence of correlated features into the best subset. The cross correlation coefficient between features is considered in order to exclude features which are correlated with the top-ranked. This technique is described in detail in [43]. Using this procedure we are able to define the maximum number of the *l* best features.

Both of the adopted FS methods measure the classification capability of a specific feature with respect to a two-class problem. Since our dataset consists of 4 classes, in order to perform the FS task, we adopted a similar to the “one against one” multi-class classification strategy. Thus, we split the dataset into 6 splits, one for each pair of classes; then we applied the 2 mentioned FS techniques to each split and lastly we explored the common top-ranked features between the 12 best feature subsets returned by the two FS techniques. Ultimately, these *l* common top-ranked features form the best feature subset which was used for the development of the presented system.

### 2.3. Classification/Prediction Models and System's Architecture

The development of an intelligent clinical decision support system involves the employment of several machine learning methods and pattern recognition/classification techniques. Machine learning is concerned about the development and the study of intelligent systems that can learn from data, while pattern recognition/classification aims to use these systems in order to classify an object into a correct class based on features characterising the object. These features are the ones provided by the FS task. The machine learning algorithms that implement pattern classification are known as classifiers. There is a variety of classifiers used for pattern classification; each one of those has certain limitations and advantages. There are simple classifiers like the *k-*nearest neighbours (*k*-NN) and the Bayesian classifier and more complex ones like the artificial neural networks (ANNs) [27, 43, 46, 47]. For example, the *k*-NN may be considered as a simple classifier because it classifies a sample based on the *k*-closest training samples in the feature space; it just computes the distances between the new sample and the samples of the training set and according to these distances it classifies the sample to the class of the closest training samples. On the contrary, ANNs are complex networks of artificial neurons interconnected with each other, which obtain knowledge and the ability to classify a sample by the application of complex learning algorithms. This capability of learning from a certain dataset makes the neural networks suitable for classification and prediction tasks in practical situations. Furthermore, neural networks are inherently nonlinear which makes them more suitable for processing complex data patterns, in contrast to many traditional methods.

In this study, in order to develop the proposed CDSS, we employed and tested 6 classifiers: the *k*-nearest neighbours (*k*-NN) classifier [46], the naïve Bayesian (NB) classifier [43], the classification and regression tree (CART) [48], and 3 types of ANNs, namely, the multilayer perceptron network (MLP) [47], the radial basis function network (RBF) [47], and the probabilistic neural network (PNN) [49, 50].

The classifiers were designed to classify the cases into the following 4 groups corresponding to the cervical histology: negative, CIN1, CIN2/3, and cancer (SCC or Adeno-Ca). The feature subset characterising each case, which is derived by the FS task, was used as the input of each classifier. Thus, each classifier takes as inputs results from the examinations of each case and outputs its classification group, providing in this way a prediction regarding the actual cervical status of each woman. The available dataset (Table 1) was divided into 3 sets: the training set (486 cases) which was used to build and train the classifiers, the validation set (126 cases) which was used to optimise the parameters of each classifier, and the test set (128 cases) which was used to evaluate their predictive performance. The 3 sets were properly stratified so that the classes' distribution in each set is approximately the same as that in the initial dataset. Thus, each set contains representative samples of the same larger population. We have also to note that, due to the many different classes and the diversity of the examinations' results, we had to use a large set for training (66% of the initial dataset) so as to provide to the classifiers representative samples of every situation. During the training phase, the cases of the training set are provided to the classifiers along with their classes (their actual histology) and the classifiers learn from this specific dataset using a learning algorithm. The cases of the validation and the test sets are not used during training; thus these cases are unknown to the classifiers (unseen data). The validation set is used as a test platform for fine tuning model's parameters and selecting the best-performing model, while the test set is used to assess the predictive performance of the developed models on data which have not been used in any way in the designing process.

Through a process of designing, training, and testing the aforementioned classifiers, we tried to investigate the potential role of these models to improve the accuracy of diagnosis. However, no single classifier produced satisfactory results. By thoroughly investigating the classification results for each case separately, we discovered that the PNN demonstrated good predictive performance for most cases with cytology LSIL and above, and specifically for women with cytology LSIL harbouring CIN grade 2 or worse (CIN2+). However, the PNN showed poor performance on identifying the correct histology of women with ASCUS cytology. On the other hand, we discovered that the MLP, even though underperformed regarding the whole dataset, produced good prediction outcomes for the cases with ASCUS cytology. This fact led us to design a hybrid architecture, by combining a PNN and a MLP.

**Figure 1:**
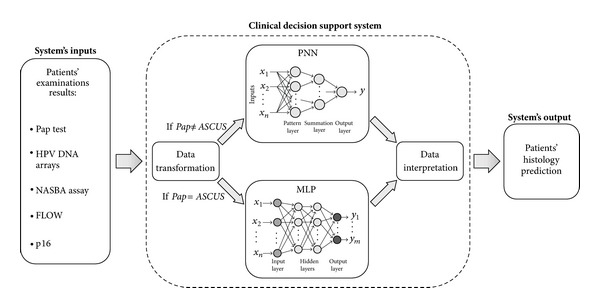
Schematic block diagram of the proposed decision support system. The examinations' results of each patient are used as inputs to the CDSS. The medical information is transformed to data appropriate for processing by the PNN and the MLP subsystems. According to the value of the Pap test, the transformed data of each case is promoted to the PNN or the MLP subsystem; if Pap test is ASCUS, the data are promoted to the MLP; otherwise they are promoted to the PNN. The output of each network is properly transformed by the data interpretation block to medical information. At the end, the CDSS provides predictions regarding the actual cervical status of each woman. NASBA: nucleic acid sequence based amplification for the identification of E6/E7 mRNA of the HPV types 16, 18, 31, 33, and 45; FLOW: flow cytometric E6/E7 HPV mRNA assay; p16: p16 immunocytochemical examination; ASCUS: atypical squamous cells of unknown significance; PNN: probabilistic neural network; MLP: multilayer perceptron network.

The presented CDSS consists of two subsystems: the main subsystem is a PNN, while the secondary is a MLP. The PNN is used for the management of all the cases excluding those with Pap test ASCUS, while the MLP is used for the management of the ASCUS cases only. According to the value of the Pap test, each case is promoted to the main or the secondary subsystem; if Pap test is ASCUS, the data are promoted to the MLP; otherwise they are promoted to the PNN. The schematic diagram of the proposed decision support system is presented in Figure 1. This architecture proved to provide better classification results comparing to a single classifier approach.

The learning and the predictive ability of an ANN is determined by several factors, such as the type of the network and the parameters of the specific type, the network's architecture (topology), the learning algorithm chosen for training, and the characteristics of the data provided to the network. Therefore, in order to construct the CDSS, the task of identifying the optimal architecture and parameters of the PNN and the MLP has to be accomplished.

#### 2.3.1. The Probabilistic Neural Network (PNN)

A probabilistic neural network is a kind of multilayer feed-forward radial basis artificial neural network suitable for classification and prediction problems. PNNs are widely used for pattern recognition problems, nonlinear mapping, and estimation of the probability of class membership and likelihood ratios. The PNN is based on the theory of Bayesian classification and is closely related to classical estimators for probability density functions [49, 50]. The basic operation performed by the PNN is an estimation of the probability density function of each class from the provided training samples using Gaussian kernels. These estimated densities are then used in a decision rule to perform the classification.

**Figure 2:**
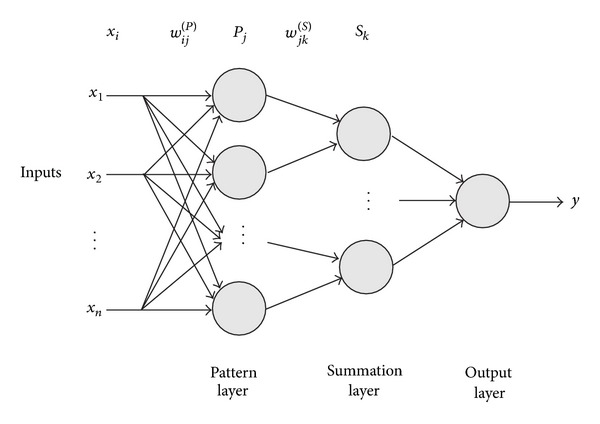
Architecture of a probabilistic neural network (PNN).

The PNN architecture consists of four layers: the input layer, the pattern layer, the summation layer, and the output layer, as depicted in Figure 2. The number of neurons of the two hidden layers (pattern and summation layers) is determined by the training set. The pattern layer contains one neuron for each sample of the training set, while the summation layer contains one neuron for each class of the training set. The training process of the PNN is straightforward and it is accomplished by setting the weights of the network using the samples of the training set; thus no learning algorithm is applied during PNN's implementation. The weights between the input and the pattern layer are set as follows:

(1) $$\begin{array}{c} w_{ij}^{\left(P\right)}=p_{ij}, \end{array}$$

where *w*_*ij*_^(*P*)^ is the weight between the *i*th neuron of the input layer and *j*th neuron of the pattern layer, and *p*_*ij*_ is the value of the *i*th feature of the *j*th sample of the training set. The weights between the pattern and the summation layer are set as follows:

(2) $$\begin{array}{c} w_{jk}^{\left(S\right)}=\{\begin{array}{cc} 1 & \text{if}\;\;T_{k}^{\left(j\right)}=1\\ 0 & \text{else}, \end{array} \end{array}$$

where *w*_*jk*_^(*S*)^ is the weight between the *j*th neuron of the pattern layer and *k*th neuron of the summation layer. The value of *T*_*k*_^(*j*)^ is 1 only when sample *j* is associated with class *k* and 0 elsewhere.

When an input is presented, the pattern layer computes the distances between the input vector and the training vectors and produces a vector which indicates how close the input is to the training samples, as follows:

(3) $$\begin{array}{c} d_{j}^{\left(P\right)}=\sqrt{\underset{i}{\sum }{\left(w_{ij}^{\left(P\right)}-x_{i}\right)}^{2}}, \end{array}$$

where *d*_*j*_^(*P*)^ is the distance between the input vector and the *j*th sample of the training set, while *x*_*i*_ denotes the *i*th variable of the input (*i*th node of input layer).

The transfer function of the neurons of the pattern layer is a radial basis function. The output of each pattern neuron is computed as

(4) $$\begin{array}{c} P_{j}=\exp\left(-\frac{d_{j}^{\left(P\right)}}{2\sigma^{2}}\right), \end{array}$$

where *σ* is a smoothing parameter corresponding to the standard deviation of the Gaussian distribution.

Each summation neuron sums the contributions for each class of the input to produce at the net output a vector of probabilities. The output of each summation node can be expressed as

(5) $$\begin{array}{c} S_{k}=\frac{1}{\sum_{j}w_{jk}^{\left(S\right)}}\underset{j}{\sum }w_{jk}^{\left(S\right)}\cdot P_{j}. \end{array}$$

Finally, a competitive transfer function in the output layer (single neuron) classifies the input vector into a specific class if that class had the maximum output value from the corresponding neuron at the summation layer:

(6) $$\begin{array}{c} y=\arg\underset{k}{\max\;}S_{k}. \end{array}$$

From the above, it is obvious that in the PNN architecture the number of the hidden layers and the transfer functions of the neurons are predefined and the number of the neurons of each hidden layer depends on the size and the form of the training set. The single free parameter of this network is the smoothing parameter sigma (*σ*), the standard deviation of the Gaussians. Thus, the selection of the optimal PNN, which will constitute the main subsystem of the CDSS, is essentially the act of determining the optimal sigma value.

#### 2.3.2. The Multilayer Perceptron Network (MLP)

The multilayer perceptron is the most widely used neural network. It is a feed-forward ANN with an input layer that receives external inputs, one or more hidden layers and an output layer providing the output of the network. Each layer of the MLP includes one or more neurons directionally linked with the neurons from the previous and the next layer. Determining the right architecture of a MLP is the task of selecting the optimal parameters of the network, such as the number of the hidden layers and the number of the neurons of each hidden layer. As far as the learning algorithm is concerned, the back-propagation (BP) algorithm [47] is the most common learning technique for training a typical MLP. During training with the BP algorithm, information about the errors of the network on known data is propagated backwards from the output layer to the input layer and it is used to adjust the connections between the layers and their neurons (the weights and biases of the network) in order to minimize the error and thus improve performance.

As far as the training of the MLPs is concerned, the Levenberg-Marquardt BP algorithm was used for the learning process, while the mean squared normalized error (MSE) was used as the network's cost function [47]. Training a MLP is essentially the process of modifying the weights and biases of the network in order to minimize this cost function. The learning rate and the momentum of the BP algorithm were set equal to 0.1 and 0.9, respectively. In order to avoid overfitting, an early-stopping learning technique was implemented, according to which the classification error on the validation subset was monitored during the training process. When the validation error increases for a specified number of iterations of the BP algorithm, the training is stopped, and the weights and biases at the minimum of the validation error are returned. For the training process, criteria for convergence were met with 40 maximum validation failures or when MSE ≤ 0.0001 or when a maximum of 1000 iterations (epochs) was reached.

As discussed in [51], empirical studies often found that networks with many hidden layers generally perform no better, and often worse, than neural networks with one or two hidden layers. Thus, in this study, we considered MLP architectures with one or two hidden layers. All neurons in the hidden and the output layers use the sigmoidal activation function. In order to identify the optimal network topology, we applied a trial-and-error cascade constructive process by adding neurons to the hidden layers, one at time, and evaluating the developed MLPs. For each MLP architecture (one-hidden-layered and two-hidden-layered), this process was stopped when the MLPs showed continuous impaired performance.

With the determined number of hidden layers and neurons, both the learning rate and the momentum coefficient of the BP algorithm were further investigated to ensure a high probability of global network convergence.

#### 2.3.3. Selection of the Optimal Models

In order to identify the optimal parameters of the developed classifiers and eventually select the optimal PNN and MLP which comprise the CDSS's subsystems, we performed the parameter tuning task by evaluating the developed models on the validation set. However, during this task we discovered that classifiers with different parameters presented the same best performance on the validation set, making it difficult to select the optimal models. Thus, in order to perform more accurately the model selection task, we also took into consideration the classification performance on the training set.

Let the classification error on the training set (resubstitution error) be denoted as *ε* ^*r*^ and the classification error on the validation set as *ε* ^*v*^. For each classification algorithm used in this study, we build *S* classifiers with different parameters, and we define as optimal the classifier which minimizes the cost function:

(7) $$\begin{array}{c} J_{m}^{}=\frac{\varepsilon_{m}^{r}+\varepsilon_{m}^{v}}{2},\;\forall m\in S,\;\;\forall \varepsilon_{m}^{v}\equiv \underset{}{\min}\left\{\varepsilon_{m}^{v}\right\}. \end{array}$$

Utilizing the above cost function for the selection of the optimal parameters, we ensure that the optimal models demonstrate the best predictive performance on the validation set (the models which minimize the cost function must produce the minimum classification error on the validation set) and at the same time they perform well on the cases of the training set (the models which minimize the cost function must produce low resubstitution error).

#### 2.3.4. Performance Evaluation

The final performance evaluation task was carried out using resubstitution and holdout validations. In resubstitution validation, the model is tested on the data which have been used in the learning process, that is, the data of the training set. This method provides a measure of the network's learning ability; yet it is not preferable for performance evaluation tasks as it is known to be optimistically biased. However, as shown in [52, 53], in discrete classification problems with large-sample categorical datasets, like the classification problem of this study, resubstitution can be significantly accurate relative to more complex error estimation schemes, since the optimistic bias and the variance of the method tend to be vanished as the sample size increases, provided that classifier complexity is not too high. For this reason we decided to take into consideration the performance of the final models when for testing the training and the validation sets are used. In holdout validation the optimal models are tested on data that were not used in any way in the building process (training and parameter tuning), that is, the data of the test set. The holdout classification error serves as a measure of the model's predictive (generalization) ability.

We have to note that we did not use complex error estimation schemes like the *k*-fold cross validation, so as to be able to study the classification result for each case separately and thus to evaluate the system at individual level. It must be noted that for the clinicians the significance of such a system stands on its ability to correctly identify cases with conflicting tests' results which are difficult to be evaluated by them. Hence, more than the overall accuracy, what is important is the correct identification of as many as possible women with insignificant cytological findings harbouring CIN2+ lesions, as well as the correct identification of women with HSIL+ cytology but with actual histology below CIN2.

Due to the above, the predictive performance of the finally selected ANNs is presented by confusion matrices obtained through testing the networks on the training, validation, and test sets.

## 3. Results

### 3.1. Feature Selection

As described, 12 feature subsets have been produced by the application of the adopted FS techniques to the split datasets, 6 of them corresponding to the ROC FS method and 6 to the MRMR FS framework. Incorporating cross correlation information into the ranking procedure of the ROC FS method, we discovered that, from the 46 variables obtained from the 5 medical tests considered, only 24 contain useful uncorrelated information. Investigating the common top-ranked uncorrelated features between the 12 subsets, we concluded that the following 18 contribute importantly to the prediction of the underlying condition of each case: Pap test, HPV-16, HPV-18, HPV-31, HPV-33, HPV-45, HPV-51, HPV-53, HPV-58, HR-HPV DNA, LR-HPV DNA, N16, N18, N31, N33, N45, FLOW, and p16. Since the attributes HPV-16, HPV-18, HPV-31, HPV-33, HPV-45, HPV-51, HPV-53, and HPV-58 correspond to high-risk subtypes of the HPV DNA test, we properly transformed the attribute HR-HPV DNA so as to correspond to the existence of the rest high-risk subtypes only. This process took place in order to dismiss the correlation between the HR-HPV DNA attribute and the rest. Eventually, these 18 features form the best feature subset characterizing each patient. Therefore, for each case, the classifiers take as inputs the values of these 18 variables.

### 3.2. Classification/Prediction Models Selection and Performance Evaluation

#### 3.2.1. Single Classifier Approach

As discussed previously, firstly we explored the single classifier approach, according to which a single classifier is being used for the management of all the cases. Six different classifiers have been developed and evaluated.  Table 3 presents the classification accuracy on the training, validation, and test sets of each model, along with its optimal parameters. It seems that the single classifier approach is not adequate to address the problem, as none of these classifiers produced satisfactory results. However, by investigating the classification results for each case separately, we discovered that, between these 6 classifiers, the PNN was superior in classifying correctly those cases with Pap test LSIL and above, whereas the MLP was superior in classifying correctly those cases with Pap test ASCUS. With regard to the cases with negative cytology, all the classifiers produced similar results with none of them performing significantly better compared to the others. Because of these findings, we designed the hybrid solution, by combining a PNN for the classification of all the cases excluding those with cytology ASCUS and a MLP for the classification of the cases with ASCUS cytology, which, according to the following results, proved to be better than the single classifier approach.

#### 3.2.2. Selection and Performance of the Optimal PNN

As mentioned before, in the PNN architecture, the only free parameter is the smoothing parameter sigma (*σ*). Thus, in order to identify the optimal network, we trained and evaluated several PNNs with different sigma values.

As the optimal PNN was used for the management of all the cases excluding those with Pap test ASCUS, in order to train and evaluate the developed PNNs, the 140 cases with ASCUS cytology were excluded from the 3 datasets; thus, 400 cases have been used for training, 100 cases for validation and 100 cases for testing these networks. The training, validation, and test sets of the PNNs are presented in Tables 4, 5, and 6, respectively.

The topology of each of the PNNs developed is described as follows: the input layer consists of 18 nodes, one for each of the features of the feature subset derived by the FS task; the pattern layer contains 400 neurons, one for each of the training samples; and the summation layer contains 4 neurons, one for each class of the training set.

Employing several PNNs with different sigma values, we concluded that performance was decreasing significantly for sigma values greater than 0.8. In order to obtain the optimal value of the parameter *σ*, and thus identify the PNN which performs best, we trained and evaluated 800 PNNs, for *σ* = 0.001 to 0.801, with a step of 0.001. It must be noted that PNNs are by design very fast networks and thus the time required to train and test 800 PNNs is not an important issue.

Evaluating the 800 developed PNNs, we concluded that the cost function *J*_*m*_ is minimized for a sigma value equal to 0.380. Thus, the optimal PNN comprising the main subsystem of the CDSS is the PNN with *σ* = 0.380. The predictive performance of this PNN is presented through confusion matrices obtained by testing the network on the training, validation, and test sets (Tables 7, 8, and 9). The overall classification accuracies of the PNN on the training, validation, and test sets are 90.0%, 82.0%, and 84.0%, respectively.

Table 10 depicts the comparison between the cytological diagnosis obtained from the Pap test and the PNN, for all the cases excluding ASCUS. Moreover, by comparing Tables 4–6 (cytological diagnosis) with Tables 7–9 (PNN's classifications), respectively, it can be observed that the PNN outperformed cytology, as it correctly classified 240 negative cases (Tables 7–9: 163 + 40 + 37), 157 CIN1 cases (Tables 7–9: 109 + 24 + 24), 111 CIN2/3 cases (Tables 7–9: 76 + 16 + 19), and 18 Ca cases (Tables 7–9: 12 + 2 + 4), comparing to the 231 negative cases (Tables 4–6: 154 + 40 + 37), 142 CIN1 cases (Tables 4–6: 97 + 23 + 22), 93 CIN2/3 cases (Tables 4–6: 62 + 15 + 16), and 15 Ca cases (Tables 4–6: 9 + 2 + 4) that cytology correctly detected. In total, the PNN predicted correctly the histology of 526 of the 600 cases, whereas cytology diagnosed correctly 481 of the 600 cases of the available dataset (excluding ASCUS). It is noteworthy that the PNN classified correctly 17 of the 27 LSIL cases harbouring CIN2/3 (Tables 4–6: 18 + 4 + 5 = 27 LSIL cases with CIN2/3 histology, Tables 7–9: 5 + 3 + 2 = 10 of these cases classified from the PNN as CIN1 and the rest 17 classified as CIN2/3) and 7 of the 22 HSIL cases with CIN1 histology (Tables 4–6: 11 + 5 + 6 = 22 HSIL cases with CIN1 histology, Tables 7–9: 7 + 4 + 4 = 15 of these cases classified from the PNN as CIN2/3 and the rest 7 classified correctly as CIN1).

#### 3.2.3. Selection and Performance of the Optimal MLP

As mentioned before, the MLP was employed exclusively for the classification of the cases with ASCUS cytology.  Table 11 shows in detail the distribution of the ASCUS cases used in the training, validation, and test sets of the MLPs.

Adopting the trial-and-error constructive process described previously, we eventually trained and evaluated 514 MLPs: 30 of them with one hidden layer, with their layer's size ranging from 10 to 40 neurons, and 484 with two hidden layers, with hidden layers' sizes ranging from 5 to 27 neurons.

Based on the experimental results, the optimal architecture of the MLP was found to be a network with two hidden layers, with 11 neurons on the first hidden layer and 17 on the second. The input layer of the MLP consists of 17 nodes, one for each of the 18 features of the feature subset excluding Pap test (as all the cases had ASCUS cytology). The output layer contains 4 neurons, one for each class of the dataset. In addition, by adopting a trial-and-error approach, the network appeared to be more efficient with the learning rate at 0.01 and the momentum at 0.8.

The overall classification accuracies of the MLP on the training, validation, and test sets are 75.6%, 76.9% and, 85.7%, respectively. Tables 12–14 present the confusion matrices of the MLP obtained through testing the network on the training, the validation, and the test sets of the ASCUS cases, respectively. Using the optimal MLP, we managed to correctly detect the actual histology of 109 of the 140 ASCUS cases (Tables 12–14). It must be noted that due to positive biomarkers, the MLP detected 9 of the 13 ASCUS cases harbouring CIN2/3 (Tables 12–14: 6 + 1 + 2).

### 3.3. Comparison between the CDSS and the Medical Tests to Detect CIN2+ Lesions

In order to evaluate the performance of the proposed CDSS compared to the tests involved in this study, we calculated the sensitivity, specificity, positive predictive value (PPV), and negative predictive value (NPV) of the methods on the basis of detecting high-grade cervical intraepithelial neoplasia and cancer (CIN2+). Moreover, we calculated Youden's index (Sensitivity+Specificity-1) of each method, which is a single statistic measure of a test's performance, used for the evaluation of the overall discriminative power of a test and for comparison of this test with others.

The performance measures have been calculated using all the cases of the dataset (740 cases). The cutoff of CIN2+ was used in order to have comparable results between the CDSS and the other medical tests. According to this threshold, the cases with histologic diagnosis of CIN1 and below were considered negative and the cases with histologic diagnosis of CIN2 and above were considered positive. The definition of positivity of each medical test is presented in Table 15. As shown is Table 15, different positivity thresholds have been taken into consideration for the Pap test and the HPV DNA test. As far as the CDSS is concerned, the values of the 18 features characterizing each patient are provided to the system and the latter classifies the case into one of the 4 groups corresponding to cervical histology. For the CDSS, positivity was defined as a classification result of CIN2/3 or cancer.  Table 16 presents the diagnostic performance of the CDSS and the medical tests, in terms of sensitivity, specificity, PPV, and NPV, in predicting high-grade cervical intraepithelial neoplasia or cancer.

In addition, we evaluated the performance of the CDSS in comparison to several combinatorial approaches of the medical tests. Two different combinatorial approaches have been taken into consideration; the “logical OR” and the “logical AND” combinations. In “OR” combinatorial approach, the combination is defined as positive when any of the combined tests is positive, while, in “AND” approach, the combination is defined as positive when all of the combined tests are positive. Tables 17, 18, 19, and 20 present the performance of the several combinations considered in detecting CIN2+ lesions.

The CDSS showed high sensitivity (89.4%), high specificity (97.1%), high PPV (89.4%), and high NPV (97.1%), for detecting CIN2+. In comparison to the medical tests involved in this study and their combinations, CDSS produced the most balanced results in terms of sensitivity, specificity, PPV, and NPV. Moreover, when ranking the tests by maximal Youden's index, which gives equal weight to sensitivity and specificity, the CDSS ranked highest (Youden's index of 0.87), outperforming all the tests and their combinations.

## 4. Discussion and Conclusions

The gynaecological smear is viewed as the most successful cancer test of all time and of all organs [54]. Nevertheless, and despite the advances of the last decade, there is still lack of consensus on the optimal management of women with abnormal pap smears; actually a proportion of women having LSIL may have a HSIL and additionally it is not infrequent that women with HSIL cytology may have CIN1 or even normal histology; finally women with ASCUS in cytology present similar problems on their management. There have been many efforts to apply various biomarkers in the triage of abnormal Pap smears [14, 19, 36, 37, 55–65]. The studied methods are either highly sensitive or highly specific, however not both at the same time and thus no perfect method is available today; in our study similar results were found (see Tables 16–20). In our material the percentage of CIN2+ cases in the total of the cases given as ASCUS is (Table 1: 13 + 1) 14/140 = 10.0%; additionally the percentage of CIN2+ cases in the total of LSIL cases is (Table 1: 27 + 2) 29/193 = 15.0% and both percentages are in agreement with these reported by other researchers in the literature [66]; specifically these are 5–17% and 9–16%, respectively. On the other hand the percentage of cases given in cytology as HSIL and being lower than CIN2 is (Table 1: 22 + 5) 27/127 = 21.26%, a percentage in agreement with the literature [67, 68].

Today, the widely accepted management options of ASCUS and LSIL smears remain either the immediate referral to colposcopy or the cytological surveillance with repeated smears. A policy of immediate referral to colposcopy could potentially result not only in the overloading of colposcopy clinics but also in overtreatment due to subtle colposcopical findings. Many young nulliparous women might be exposed to the physical and psychological sequelae of unnecessary treatment with long-term perinatal morbidity in women being in reproductive age [69–71]. On the other hand, repeating a cervical smear carries the risk of missing high-grade lesions (HSILs), increases nonattending rates (noncompliance [72]), and increases social and psychological burden of women, directly questioning organized screening programs' (OSPs) credibility. Therefore, it is essential to reduce unnecessary colposcopies and, if feasible, to have in advance indication for women treatment, even before the colposcopical examination. Thus, a methodology for more accurate diagnosis is extremely important.

Although HPV related tests may be used in the triage of ASCUS cases [65, 73], every effort should be made to develop new tools and biomarkers to improve the accuracy of diagnosis and allow tailored management. Nowadays, there are numerous methods and biomarkers that are available for cervical cancer detection; nevertheless no single method is optimal [56]. Thus, a different approach is required that will be able to combine many parameters in order to produce an accurate risk assessment for each woman. Instead of the futile search for a single golden marker we should evolve current ones and invent more elaborate methods for result evaluation and utilisation. Based on this, we are working since 2010 on an innovative approach of employing advanced mathematical and computing tools for the nonlinear combination of the methods and biomarkers that are available for cervical cancer detection. Up to now preliminary results are presented in the literature [36, 37].

The aim of this study was to create a decision support system for the triage of women before referral to colposcopy. This system is based on the standard cytological diagnosis on ThinPrep Pap test smears and the expression of various biomarkers. The preliminary results suggest that the proposed neural network architecture may improve the accuracy of diagnosis; according to Tables 16–20, CDSS provided the most balanced results in terms of specificity, sensitivity, PPV, and NPV in comparison to the medical tests involved in this study and their combinations. The cutoff of CIN2+ was used because it is the decision threshold that a case is therapeutically handled; cases below CIN2+ are strictly monitored.

In our material, regarding the underestimated cases (CIN2+ cases which were classified by the CDSS as negatives or CIN1), only 1 out of the 4 misclassified ASCUS cases (Tables 12 and 13) was CIN3 and from the 13 misclassified non-ASCUS cases (Tables 7–9); 2 were CIN3 and 1 Adeno-Ca. Especially for the one misclassified adenocarcinoma, the total of the biomarkers was negative and the case was given as LSIL in cytology. On the other hand, the case of SCC that was given as LSIL in cytology (Table 4) was correctly classified by the PNN due to the fact that there were positive biomarkers (Table 7). Moreover, it is noteworthy that the CDSS classified correctly 9 of the 13 ASCUS cases and 17 of the 27 LSIL cases harbouring CIN2/3 (as presented in the results section).

In this study, the sensitivity of cytology using ASCUS+ as a cutoff was higher than HR-HPV DNA test (see Table 16: 98.1% versus 89.4%) in contrast to other studies, such as the ATHENA study [74]. The reader should be aware that this may be caused by verification bias related to the fact that cytology positive and HPV negative women had biopsies, in contrary to cytology negative and HPV positive women with a negative colposcopy. According to other studies [74] the sensitivity of HPV DNA test is higher than the sensitivity of cytology; however, in our case a special small population for referral to colposcopy is involved, in contrast to the generic population used in the ATHENA study. In addition, the laboratory bases the cytological examination on experienced cytopathologists as reported in our previous study [56] and thus the performance of the cytological examination is higher than the standard reported performance. In another study [75], the sensitivities of LBC and HPV DNA test are comparable with our results; additionally in another study [76] lower sensitivity of the HPV DNA test than the sensitivity of the cytological examination is reported. To conclude, performing our study on meta-analysis data would be impossible as detailed information for each individual case tests' results is required in order to train and test the CDSS system; therefore, a rather small but controlled population was preferred.

A potential application of this system is to support the decision of referring a woman to colposcopy or not. A work flow scenario is as follows: the cytological examination is used as primary test and only an ASCUS+ result is followed by the application of the other four ancillary tests using the remaining material in the vial. Subsequently, the five tests' results (including cytology) serve as inputs to the CDSS for evaluation and the CDSS outcome supports the final decision making for referring to colposcopy or not. The application of all five tests in general population would be a very costly process and thus our method nowadays has the potential for application in the triage of ASCUS+ cases. However, a detailed cost/benefit, cost/effectiveness analysis is required as the cost of the tests is not the only factor that should be taken into account. Other important factors are the cost of the woman's transportation to a colposcopy clinic, especially in mountainous places, islands, or isolated cities/villages, the increment of recall time, and the psychological effects to the woman and her family among others.

In the literature there are already simpler techniques proposed for the triage of ASCUS and LSIL, such as the repeat cytology and the application of mRNA testing. As mentioned in [77–79], the use of NASBA HPV mRNA test in triage of women with ASCUS and LSIL may reduce the referral rate to colposcopy. As presented in [77], the HPV mRNA test significantly reduced the time from the first abnormal cytology until biopsy and had predictive values comparable with those of repeat cytology. In [79], the authors report that HPV mRNA testing is a better triage test for women with LSIL than repeat cytology, as it was more sensitive (94.2%) and specific (86.0%) for detecting CIN2+. In addition, the HPV mRNA test showed higher PPV (67.0%) compared to repeat cytology (38.4%). In a meta-analysis of the accuracy of mRNA testing for detecting CIN2+, the mRNA testing was substantially more specific than the HPV DNA test in women with ASCUS and LSIL [79]. However, it demonstrated lower sensitivity and thus women with negative mRNA test results cannot be considered free of CIN2+ and require followup [79]. In our study, the proposed system showed higher sensitivity, higher specificity, higher PPV, and higher NPV compared to NASBA mRNA testing, for detecting CIN2+. In comparison to the HPV DNA test, the proposed system is a little less sensitive in detecting CIN2+; however, its specificity and PPV are significantly higher. According to our results, the proposed system produced the most balanced results in terms of sensitivity, specificity, PPV, and NPV and demonstrated the highest Youden's index, compared to cytology and the biomarkers used in the study and their combinations. Thus, in comparison to the already proposed schemes for triage of ASCUS+, our approach may produce more accurate results, leading to improved triage of ASCUS+ and improved detection of CIN2+. Therefore, the overhead for both cytological laboratories and colposcopy rooms can be reduced.

The application of the proposed CDSS gave promising results, suggesting that such an approach may significantly improve the accuracy of diagnosis. Furthermore, the notable performance of the CDSS in identifying women with LSIL cytology at risk of developing cancer suggests that such systems may play an important role in triage decisions and hence may reduce the overload of colposcopy clinics and guide personalised management and therapeutic interventions. The results should be further assessed in larger datasets in order to confirm the reproducibility of these findings. As some of the tests and biomarkers may result in increased cost, our research is now directed to develop a more cost-effective CDSS which will use fewer tests, without losing much in performance. Furthermore, machine learning techniques for handling missing values are under examination, in order to be able to provide outcomes also for cases with missing or invalid examinations' results.

Today, the CDSS is available to users as a PC application. Our future work involves the upgrade of the CDSS to an intelligent web service for patient-specific prediction, progression, and prognosis of cervical cancer, available over the Internet to the worldwide medical community, which will serve as a decision support system to physicians and medical researchers for the management of new cases or the followup of existing cases.

## Figures and Tables

**Table 1:** Correlation between the cytological and histological findings of the dataset used in the study.

	Pap test result					Total
	WNL	ASCUS	LSIL	HSIL	SCC/Adeno-Ca	
Histological examination result						
Clinically negative	196	0	0	0	0	196 (26.5%)
Negative	35	60	22	5	0	122 (16.5%)
CIN1	31	66	142	22	0	261 (35.3%)
CIN2/3	3	13	27	93	0	136 (18.4%)
SCC/Adeno-Ca	0	1	2	7	15	25 (3.4%)

Total	265 (35.8%)	140 (18.9%)	193 (26.1%)	127 (17.2%)	15 (2%)	740

WNL: within normal limits, ASCUS: atypical squamous cells of unknown significance, LSIL: low-grade squamous intraepithelial lesion, HSIL: high-grade squamous intraepithelial lesion, SCC: squamous cell carcinoma, Adeno-Ca: adenocarcinoma, CIN: cervical intraepithelial neoplasia.

**Table 2:** Variables characterising patients' biological status.

Variable name	Description	Value range
Pap test	The result of the cytological examination expressed according to Bethesda system	1 : WNL, 2 : ASCUS, 3 : LSIL, 4 : HSIL, 5 : SCC or ADENO-Ca

HPV DNA Arrays: HPV-6, HPV-11, HPV-16, HPV-18, HPV-26, HPV-31, HPV-33, HPV-35, HPV-39, HPV-40, HPV-42, HPV-43, HPV-44, HPV-45, HPV-51, HPV-52, HPV-53, HPV-54, HPV-56, HPV-58, HPV-59, HPV-61, HPV-62, HPV-66, HPV-68, HPV-70, HPV-71, HPV-72, HPV-73, HPV-81, HPV-82, HPV-83, HPV-84, HPV-85, HPV-89	The existence of individual subtypes according to the HPV DNA examination	0 if the specific subtype is not found, 1 if the specific subtype is found

HR-HPV DNA	The existence of high-risk subtypes found by the HPV DNA test	0 if none of the high-risk types was found, 1 if at least one of the high-risk types is found

LR-HPV DNA	The existence of low-risk subtypes found by the HPV DNA test	0 if none of the low-risk types is found, 1 if at least one of the low-risk types is found

Arrays number	The number of HPV subtypes found by the HPV DNA test	Expressed as number

N16	The result of the NASBA mRNA test for HPV subtype 16	0 if negative, 1 if positive

N18	The result of the NASBA mRNA test for HPV subtype 18	0 if negative, 1 if positive

N31	The result of the NASBA mRNA test for HPV subtype 31	0 if negative, 1 if positive

N33	The result of the NASBA mRNA test for HPV subtype 33	0 if negative, 1 if positive

N45	The result of the NASBA mRNA test for HPV subtype 45	0 if negative, 1 if positive

FLOW	The result of the identification of E6/E7 mRNA expression of high-risk HPV using flow cytometry technique	0 if negative (positive expression <1.5%), 1 if positive (positive expression >1.5%)

p16	The result of the p16 immunocytochemical examination	0 if negative, 1 if positive

WNL: within normal limits, ASCUS: atypical squamous cells of unknown significance, LSIL: low-grade intraepithelial lesion, HSIL: high-grade squamous intraepithelial lesion, SCC: squamous cell carcinoma, ADENO-Ca: adenocarcinoma.

**Table 3:** Classification accuracies on the training, validation, and test sets of the 6 classifiers developed (single classifier approach).

Classifier	*k*-NN	NB	CART	MLP	RBF	PNN
Optimal parameters	*k* = 5	—	(i) Pruning level = 7/11 (ii) Number of terminal nodes = 8	2 hidden layers 18 × 18	(i) 486 neurons in the hidden layer (all samples of training set) (ii) Sigma = 0.6	Sigma = 0.4

Training set	78.6%	76.8%	77.6%	78.4%	87.6%	87.6%
Validation set	79.4%	80.9%	78.6%	77.0%	77.7%	80.2%
Test set	82.8%	82.8%	75.8%	80.5%	70.3%	80.0%

*k*-NN: *k*-nearest neighbours classifier, NB: naïve Bayesian classifier, CART: classification and regression tree, MLP: multilayer perceptron network, RBF: radial basis function network, PNN: probabilistic neural network.

**Table 4:** Training set of PNNs (histology and cytology).

	Pap test result				Total
	WNL	LSIL	HSIL	SCC/Adeno-Ca	
Histological examination result					
Negative/clinically negative	154	16	3	0	173 (43.3%)
CIN1	21	97	11	0	129 (32.2%)
CIN2/3	2	18	62	0	82 (20.5%)
SCC/Adeno-Ca	0	1	6	9	16 (4.0%)

Total	177 (44.2%)	132 (33.0%)	82 (20.5%)	9 (2.3%)	400

**Table 5:** Validation set of PNNs (histology and cytology).

	Pap test result				Total
	WNL	LSIL	HSIL	SCC/Adeno-Ca	
Histological examination result					
Negative/clinically negative	40	2	1	0	43 (43.0%)
CIN1	5	23	5	0	33 (33.0%)
CIN2/3	1	4	15	0	20 (20.0%)
SCC/Adeno-Ca	0	1	1	2	4 (4.0%)

Total	46 (46.0%)	30 (30.0%)	22 (22.0%)	2 (2.0%)	100

**Table 6:** Test set of PNNs (histology and cytology).

	Pap test result				Total
	WNL	LSIL	HSIL	SCC/Adeno-Ca	
Histological examination result					
Negative/clinically negative	37	4	1	0	42 (42.0%)
CIN1	5	22	6	0	33 (33.0%)
CIN2/3	0	5	16	0	21 (21.0%)
SCC/Adeno-Ca	0	0	0	4	4 (4.0%)

Total	42 (42.0%)	31 (31.0%)	23 (23.0%)	4 (4.0%)	100

**Table 7:** Confusion matrix obtained through testing the PNN on the cases of the training set (Table 4).

	PNN classification result			
	Negative	CIN1	CIN2/3	Ca
Histological examination result				
Negative/clinically negative	163	10	0	0
CIN1	13	109	7	0
CIN2/3	1	5	76	0
SCC/Adeno-Ca	0	0	4	12

**Table 8:** Confusion matrix obtained through testing the PNN on the cases of the validation set (Table 5).

	PNN classification result			
	Negative	CIN1	CIN2/3	Ca
Histological examination result				
Negative/clinically negative	40	2	1	0
CIN1	5	24	4	0
CIN2/3	1	3	16	0
SCC/Adeno-Ca	0	1	1	2

**Table 9:** Confusion matrix obtained through testing the PNN on the cases of the test set (Table 6).

	PNN classification result			
	Negative	CIN1	CIN2/3	Ca
Histological examination result				
Negative/clinically negative	37	5	0	0
CIN1	5	24	4	0
CIN2/3	0	2	19	0
SCC/Adeno-Ca	0	0	0	4

**Table 10:** Diagnostic accuracy of cytology and the PNN for all the cases excluding ASCUS.

	Cytological diagnosis			PNN diagnosis		
	Training set	Validation set	Test set	Training set	Validation set	Test set
Histology						
Negative/clinically negative	89.0%	93.0%	88.1%	94.2%	93.0%	88.1%
CIN1	75.2%	69.7%	66.7%	84.5%	72.7%	72.7%
CIN2/3	75.6%	75.0%	76.2%	92.7%	80.0%	90.5%
SCC/Adeno-Ca	56.3%	50.0%	100.0%	75.0%	50.0%	100.0%
Average accuracy per set	80.5%	80.0%	79.0%	90.0%	82.0%	84.0%
Overall accuracy	**80.2% (481/600 cases)**			**87.7% (526/600 cases)**		

**Table 11:** Training, validation, and test sets of the MLPs (ASCUS cases only).

	Training set	Validation set	Test set	Total
Histological examination result				
Negative	36	12	12	60 (42.8%)
CIN1	40	12	14	66 (47.2%)
CIN2/3	9	2	2	13 (9.3%)
SCC/Adeno-Ca	1	0	0	1 (0.7%)

Total	86 (61.4%)	26 (18.6%)	28 (20.0%)	140

**Table 12:** Confusion matrix obtained through testing the MLP on the training set of the ASCUS cases.

	MLP classification result			
	Negative	CIN1	CIN2/3	Ca
Histological examination result				
Negative/clinically negative	34	2	0	0
CIN1	14	25	1	0
CIN2/3	2	1	6	0
SCC/Adeno-Ca	0	0	1	0

**Table 13:** Confusion matrix obtained through testing the MLP on the validation set of the ASCUS cases.

	MLP classification result			
	Negative	CIN1	CIN2/3	Ca
Histological examination result				
Negative/clinically negative	11	1	0	0
CIN1	4	8	0	0
CIN2/3	0	1	1	0
SCC/Adeno-Ca	0	0	0	0

**Table 14:** Confusion matrix obtained through testing the MLP on the test set of the ASCUS cases.

	MLP classification result			
	Negative	CIN1	CIN2/3	Ca
Histological examination result				
Negative/clinically negative	11	1	0	0
CIN1	3	11	0	0
CIN2/3	0	0	2	0
SCC/Adeno-Ca	0	0	0	0

**Table 15:** Definition of positivity of the medical tests involved in this study for performance evaluation purposes.

Medical tests	Definition of positivity
Pap test (cut-off ASCUS+)	ASCUS or worse
Pap test (cut-off LSIL+)	LSIL or worse
Pap test (cut-off HSIL+)	HSIL or worse
HPV DNA test	Existence of any HPV subtype found by the HPV DNA test
HR-HPV DNA	Existence of at least one of the high-risk subtypes found by the HPV DNA test
NASBA E6/E7 HPV mRNA test	Positive result of the E6/E7 HPV mRNA test (NASBA) for any of the HPV subtypes 16, 18, 31, 33, and 45
Flow cytometric E6/E7 HPV mRNA assay	Positive result of the identification of E6/E7 mRNA expression of high-risk HPV using flow cytometry technique (positive expression >1.5%)
p16	Positive result of the p16 immunocytochemical examination

Different positivity thresholds have been taken into consideration for Pap test and HPV DNA test. HR-HPV: high-risk human papillomavirus, ASCUS: atypical squamous cells of unknown significance, LSIL: low-grade intraepithelial lesion, and HSIL: high-grade squamous intraepithelial lesion.

**Table 16:** Diagnostic performance of cytology, biomarkers, and the CDSS to identify high-grade cervical intraepithelial neoplasia or cancer (CIN2+).

Histology endpoint CIN2+	Sensitivity (%)	Specificity (%)	PPV (%)	NPV (%)	Youden's index
Pap test (cut-off ASCUS+)	98.1	45.3	33.3	98.9	0.43
Pap test (cut-off LSIL+)	89.4	67.0	43.0	96.0	0.56
Pap test (cut-off HSIL+)	71.4	95.3	81.0	92.3	0.67
HPV DNA test	91.9	61.5	39.9	96.5	0.53
HR-HPV DNA	89.4	67.4	43.2	95.8	0.57
NASBA E6/E7 HPV mRNA test	77.0	90.2	68.5	93.4	0.67
Flow cytometric E6/E7 HPV mRNA assay	93.2	81.9	58.8	97.7	0.75
p16	58.4	92.9	69.6	88.9	0.51
CDSS	89.4	97.1	89.4	97.1	0.87

Statistical measures have been calculated using all the cases of the dataset (Table 1). Histology endpoint is CIN2+ for all cases. Definition of positivity of each medical test is presented in Table 15. For the CDSS, positivity was defined as a classification result of CIN2/3 or cancer. CIN2+: cervical intraepithelial neoplasia grade 2 or worse, PPV: positive predictive value, and NPV: negative predictive value.

**Table 17:** Performance of type “OR” combinations between two tests in detecting CIN2+.

Combinations of medical tests	Cytology cutoff	Sensitivity (%)	Specificity (%)	PPV (%)	NPV (%)	Youden's index
Pap test or HPV DNA	ASCUS+	99.4	37.5	30.7	99.5	0.37
Pap test or HPV DNA	LSIL+	96.3	52.0	35.8	98.0	0.48
Pap test or HPV DNA	HSIL+	96.3	60.3	40.3	98.3	0.57
Pap test or HR-HPV DNA	ASCUS+	98.8	39.2	31.1	99.1	0.38
Pap test or HR-HPV DNA	LSIL+	95.7	54.7	37.0	97.8	0.50
Pap test or HR-HPV DNA	HSIL+	95.7	65.8	43.8	98.2	0.62
Pap test or NASBA	ASCUS+	98.8	44.2	33.0	99.2	0.43
Pap test or NASBA	LSIL+	94.4	64.6	42.6	97.7	0.59
Pap test or NASBA	HSIL+	88.8	87.4	66.2	96.6	0.76
Pap test or FLOW	ASCUS+	99.4	41.8	32.2	99.6	0.41
Pap test or FLOW	LSIL+	97.5	61.7	41.4	98.9	0.59
Pap test or FLOW	HSIL+	96.9	80.3	57.8	98.9	0.77
Pap test or p16	ASCUS+	99.4	45.3	33.5	99.6	0.45
Pap test or p16	LSIL+	92.5	66.1	43.2	97.0	0.59
Pap test or p16	HSIL+	81.4	90.2	69.7	94.6	0.72
HPV DNA or NASBA		93.8	60.3	39.6	97.2	0.54
HPV DNA or FLOW		98.1	56.6	38.6	99.1	0.55
HPV DNA or p16		93.8	59.6	39.2	97.2	0.53
HR-HPV DNA or NASBA		91.9	65.8	42.8	96.7	0.58
HR-HPV DNA or FLOW		97.5	61.5	41.3	98.9	0.59
HR-HPV DNA or p16		92.5	64.8	42.2	96.9	0.57
NASBA or FLOW		96.3	79.6	56.8	98.7	0.76
NASBA or p16		87.0	85.7	62.8	95.9	0.73
FLOW or p16		96.3	77.5	54.4	98.7	0.74

Statistical measures have been calculated using all cases of the dataset (Table 1). Histology endpoint is CIN2+ for all cases. Definition of positivity of each medical test is presented in Table 15. CIN2+: cervical intraepithelial neoplasia grade 2 or worse, HR-HPV: high-risk human papillomavirus, NASBA: nucleic acid sequence based amplification for the identification of E6/E7 mRNA of the HPV types 16, 18, 31, 33, and 45, FLOW: flow cytometric E6/E7 HPV mRNA assay, PPV: positive predictive value, and NPV: negative predictive value.

**Table 18:** Performance of several type “OR” combinations between more than two tests in detecting CIN2+.

Combinations of medical tests	Cytology cutoff	Sensitivity (%)	Specificity (%)	PPV (%)	NPV (%)	Youden's index
Pap test or HPV DNA or NASBA	ASCUS+	99.4	36.6	30.4	99.5	0.36
Pap test or HPV DNA or NASBA	LSIL+	96.3	51.1	35.4	98.0	0.47
Pap test or HPV DNA or NASBA	HSIL+	96.3	59.1	39.5	98.3	0.55
Pap test or HPV DNA or FLOW	ASCUS+	100.0	35.1	30.0	100.0	0.35
Pap test or HPV DNA or FLOW	LSIL+	98.1	48.9	34.8	99.0	0.47
Pap test or HPV DNA or FLOW	HSIL+	98.1	56.0	38.3	99.1	0.54
Pap test or HPV DNA or p16	ASCUS+	99.4	37.5	30.7	99.5	0.37
Pap test or HPV DNA or p16	LSIL+	96.3	51.3	35.5	98.0	0.48
Pap test or HPV DNA or p16	HSIL+	96.3	58.9	39.4	98.3	0.55
Pap test or HPV DNA or NASBA or FLOW	ASCUS+	100.0	34.9	29.9	100.0	0.35
Pap test or HPV DNA or NASBA or FLOW	LSIL+	98.1	48.7	34.7	98.9	0.47
Pap test or HPV DNA or NASBA or FLOW	HSIL+	98.1	55.4	38.0	99.1	0.54
Pap test or HPV DNA or NASBA or FLOW or p16	ASCUS+	100.0	34.9	29.9	100.0	0.35
Pap test or HPV DNA or NASBA or FLOW or p16	LSIL+	98.1	48.0	34.4	98.9	0.46
Pap test or HPV DNA or NASBA or FLOW or p16	HSIL+	98.1	54.4	37.4	99.1	0.53
HPV DNA or NASBA or FLOW		98.1	56.1	38.3	99.1	0.54
HPV DNA or NASBA or p16		93.8	58.5	38.6	97.1	0.52
HPV DNA or NASBA or FLOW or p16		98.1	54.7	37.6	99.1	0.53
NASBA or FLOW or p16		98.1	76.7	53.9	99.3	0.75

Statistical measures have been calculated using all cases of the dataset (Table 1). Histology endpoint is CIN2+ for all cases. Definition of positivity of each medical test is presented in Table 15. CIN2+: cervical intraepithelial neoplasia grade 2 or worse, HR-HPV: high-risk human papillomavirus, NASBA: nucleic acid sequence based amplification for the identification of E6/E7 mRNA of the HPV types 16, 18, 31, 33, and 45, FLOW: flow cytometric E6/E7 HPV mRNA assay, PPV: positive predictive value, and NPV: negative predictive value.

**Table 19:** Performance of type “AND” combinations between two tests in detecting CIN2+.

Combinations of medical tests	Cytology cutoff	Sensitivity (%)	Specificity (%)	PPV (%)	NPV (%)	Youden's index
Pap test and HPV DNA	ASCUS+	90.7	69.3	45.1	96.4	0.60
Pap test and HPV DNA	LSIL+	85.1	76.5	50.2	94.9	0.62
Pap test and HPV DNA	HSIL+	67.1	96.5	84.4	91.3	0.64
Pap test and HR-HPV DNA	ASCUS+	88.8	73.4	48.1	95.9	0.62
Pap test and HR-HPV DNA	LSIL+	83.2	79.6	53.2	94.5	0.63
Pap test and HR-HPV DNA	HSIL+	65.2	96.9	85.4	90.9	0.62
Pap test and NASBA	ASCUS+	76.4	91.2	70.7	93.3	0.68
Pap test and NASBA	LSIL+	72.0	92.6	73.0	92.3	0.65
Pap test and NASBA	HSIL+	59.6	98.1	89.7	89.7	0.58
Pap test and FLOW	ASCUS+	91.9	85.3	63.5	97.4	0.77
Pap test and FLOW	LSIL+	85.1	87.2	64.9	95.5	0.72
Pap test and FLOW	HSIL+	67.7	96.9	85.8	91.5	0.65
Pap test and p16	ASCUS+	57.1	92.9	69.2	88.6	0.50
Pap test and p16	LSIL+	55.3	93.8	71.2	88.3	0.49
Pap test and p16	HSIL+	48.4	98.1	87.6	87.3	0.47
HPV DNA and NASBA		75.2	91.4	70.8	93.0	0.67
HPV DNA and FLOW		87.0	86.7	64.5	96.0	0.74
HPV DNA and p16		56.5	94.8	75.2	88.7	0.51
HR-HPV DNA and NASBA		74.5	91.7	71.4	92.8	0.66
HR-HPV DNA and FLOW		85.1	87.7	65.9	95.5	0.73
HR-HPV DNA and p16		55.3	95.5	77.4	88.5	0.51
NASBA and FLOW		73.9	92.4	73.0	92.7	0.66
NASBA and p16		48.4	97.4	83.9	87.2	0.46
FLOW and p16		55.3	97.2	84.8	88.7	0.53

Statistical measures have been calculated using all cases of the dataset (Table 1). Histology endpoint is CIN2+ for all cases. Definition of positivity of each medical test is presented in Table 15. CIN2+: cervical intraepithelial neoplasia grade 2 or worse, HR-HPV: high-risk human papillomavirus, NASBA: nucleic acid sequence based amplification for the identification of E6/E7 mRNA of the HPV types 16, 18, 31, 33, and 45, FLOW: flow cytometric E6/E7 HPV mRNA assay, PPV: positive predictive value, and NPV: negative predictive value.

**Table 20:** Performance of several type “AND” combinations between more than two tests in detecting CIN2+.

Combinations of medical tests	Cytology cutoff	Sensitivity (%)	Specificity (%)	PPV (%)	NPV (%)	Youden's index
Pap test and HPV DNA and NASBA	ASCUS+	74.5	91.5	71.0	92.8	0.66
Pap test and HPV DNA and NASBA	LSIL+	70.2	92.9	73.4	91.8	0.63
Pap test and HPV DNA and NASBA	HSIL+	57.8	98.1	89.4	89.3	0.56
Pap test and HPV DNA and FLOW	ASCUS+	86.3	87.7	66.2	95.8	0.74
Pap test and HPV DNA and FLOW	LSIL+	80.7	88.9	67.0	94.3	0.70
Pap test and HPV DNA and FLOW	HSIL+	63.4	97.4	87.2	90.5	0.61
Pap test and HPV DNA and p16	ASCUS+	55.3	94.8	74.8	88.4	0.50
Pap test and HPV DNA and p16	LSIL+	53.4	95.0	74.8	88.0	0.48
Pap test and HPV DNA and p16	HSIL+	46.6	98.6	90.4	86.9	0.45
Pap test and HPV DNA and NASBA and FLOW	ASCUS+	71.4	93.1	74.2	92.1	0.65
Pap test and HPV DNA and NASBA and FLOW	LSIL+	67.1	94.0	75.5	91.1	0.61
Pap test and HPV DNA and NASBA and FLOW	HSIL+	55.3	98.4	90.8	88.8	0.54
Pap test and HPV DNA and NASBA and FLOW and p16	ASCUS+	44.7	98.8	91.1	86.5	0.44
Pap test and HPV DNA and NASBA and FLOW and p16	LSIL+	43.5	98.8	90.9	86.3	0.42
Pap test and HPV DNA and NASBA and FLOW and p16	HSIL+	38.5	99.5	95.4	85.3	0.38
HPV DNA and NASBA and FLOW		72.0	93.1	74.4	92.3	0.65
HPV DNA and NASBA and p16		46.6	97.6	84.3	86.8	0.44
HPV DNA and NASBA and FLOW and p16		45.3	98.8	91.3	86.7	0.44
NASBA and FLOW and p16		47.2	98.8	91.6	87.1	0.46

Statistical measures have been calculated using all cases of the dataset (Table 1). Histology endpoint is CIN2+ for all cases. Definition of positivity of each medical test is presented in Table 15. CIN2+: cervical intraepithelial neoplasia grade 2 or worse, HR-HPV: high-risk human papillomavirus, NASBA: nucleic acid sequence based amplification for the identification of E6/E7 mRNA of the HPV types 16, 18, 31, 33, and 45, FLOW: flow cytometric E6/E7 HPV mRNA assay, PPV: positive predictive value, and NPV: negative predictive value.
